# The Role of Humidity in the Management of Premature Neonates in a Rural Incubator

**DOI:** 10.7759/cureus.14411

**Published:** 2021-04-10

**Authors:** Hemmanoor Samartharam, Nagashree Vasudeva, Sai Samyuktha Ila

**Affiliations:** 1 Obstetrics and Gynaecology, Sandhya Ram Hospital, Palakkad, IND; 2 Medicine, Sandhya Ram Hospital, Palakkad, IND; 3 Obstetrics and Gynaecology, Divya Hospital, Thirpur, IND

**Keywords:** rural incubator, humidity in a rural incubator, open premature care, cost-effective neonatal care

## Abstract

Background

While growing inside the uterus, the human fetus floats in amniotic fluid, and the mother maintains a stable temperature of 37 °C and a humidity of 100%. In most neonatal incubators, a stable temperature is maintained but not the humidity. We hypothesised that maintaining a humidity of 70% and a temperature of 32 °C in incubator rooms might improve the outcomes related to low birth weight (LBW) neonates.

Methods

In this interventional study, 30 preterm LBW neonates delivered at different gestational ages were studied. Instead of an incubator box, we converted one entire room (14’/9’/10’) into an incubator. Three 200-watt bulbs were fixed to the wall at a height of 1 meter from babies. The room thermometer was mounted on the wall close to babies. The room temperature was maintained at 32 °C by turning the lights on or off as required. Wet cotton sheets (4’ × 6’) were spread on the opposite wall with the support of a stand. A hygrometer was fixed to the wall near to babies, and the humidity of the room was maintained at 70-80%.

The hydration and nutrition needs of the babies were met with IV fluids/nasogastric (NG) tube feeding. Antenatal steroids were given to all mothers before the completion of 38 weeks. Babies were discharged when they were stable, and further care was given at home with similar arrangements of maintaining temperature and humidity. Birth weights, the number of babies that developed neonatal respiratory distress syndrome (NRDS), hypothermia, septicaemia, neonatal intensive care unit (NICU) admission days, home incubator days, and neonatal deaths were recorded and compared with the findings in the existing literature.

Results

Among the 30 neonates studied, birth weights ranged from 1.00 to 1.95 kg. Twenty-three babies developed NRDS, and four babies developed septicaemia; NICU days ranged from five to 28 days, and at-home incubator days ranged from 15 to 60 days. One baby succumbed to the illness.

Conclusion

Open nursing care of functionally premature neonates at room temperature of 32 °C and humidity of 70% is a cost-effective method that can lead to excellent outcomes.

## Introduction

Premature birth (PTB) is a serious medical problem globally, and it is the leading cause of neonatal mortality and long-term morbidity [1]. As per a Lancet report published in 2012, as many as 15 million preterm births occurred worldwide in 2010. The majority of these preterm births occurred in low-resource countries in South Asia and sub-Saharan Africa [2]. The global action report by WHO, “Born Too Soon”, has stated that the incidence of preterm birth is progressively increasing worldwide [3]. In developing countries, insufficient resources and poor infrastructure are the primary causes, whereas iatrogenic late preterm birth (80%) is the prominent cause in developed countries [3]. As the cost and expertise required for premature neonatal care are high and scarce, many babies lose their lives in low-resource settings. Hence, there is an urgent need to devise and implement simpler and low-cost techniques to save these babies from early deaths. Kangaroo care of premature neonates is one such interventional technique [4].

The human fetus, while growing inside the uterus, floats in warm amniotic fluid. The mother maintains a stable temperature of 37 °C and a humidity of 100%. In most neonatal incubators, the stable temperature is generally maintained but the same cannot be said of the humidity. As part of our study, we theorised that maintaining a humidity of 70% and a temperature of 32 °C in the incubator room environment may improve the outcomes related to low birth weight (LBW) neonates.

Previous studies have shown the significant impact of ambient humidity on child health, especially related to climate-sensitive infectious diseases, diarrhoeal diseases, respiratory system diseases, and paediatric allergic diseases [5]. Children are inherently sensitive to climate change because they are physiologically and metabolically less effective at adapting to weather-related exposures. Their relatively immature immune systems put them at increased risk of serious consequences from a variety of infectious diseases [6].

In this study, we attempted to come up with a cost-effective premature neonate caring facility, which can maintain adequate humidity and temperature in low-resource settings and can be practicable even at homes. We also compared our results with findings from other studies in the literature.

## Materials and methods

Informed and written consent was obtained from all mothers who participated in this study. Consent was also taken to use photographs and videos of the infants for scientific publication (sample consent form shown in Figure 4, Appendix section). The study adhered to the standards set by the Declaration of Helsinki.

**Figure 1:**
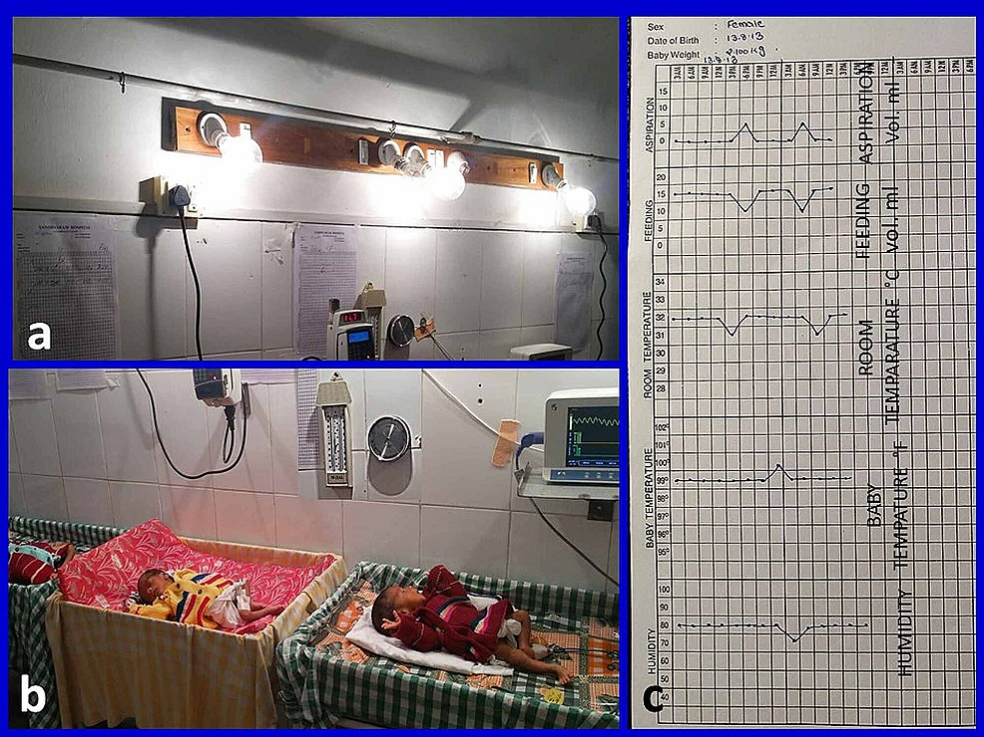
Rural incubator images (a and b): three 200-watt bulbs, a room thermometer, and a hygrometer are fixed on the wall. Three babies wearing sweaters are seen; (c) chart showing room temperature, babys' temperature, humidity, feed volume, and aspiration fluid volume from the stomach

**Figure 2:**
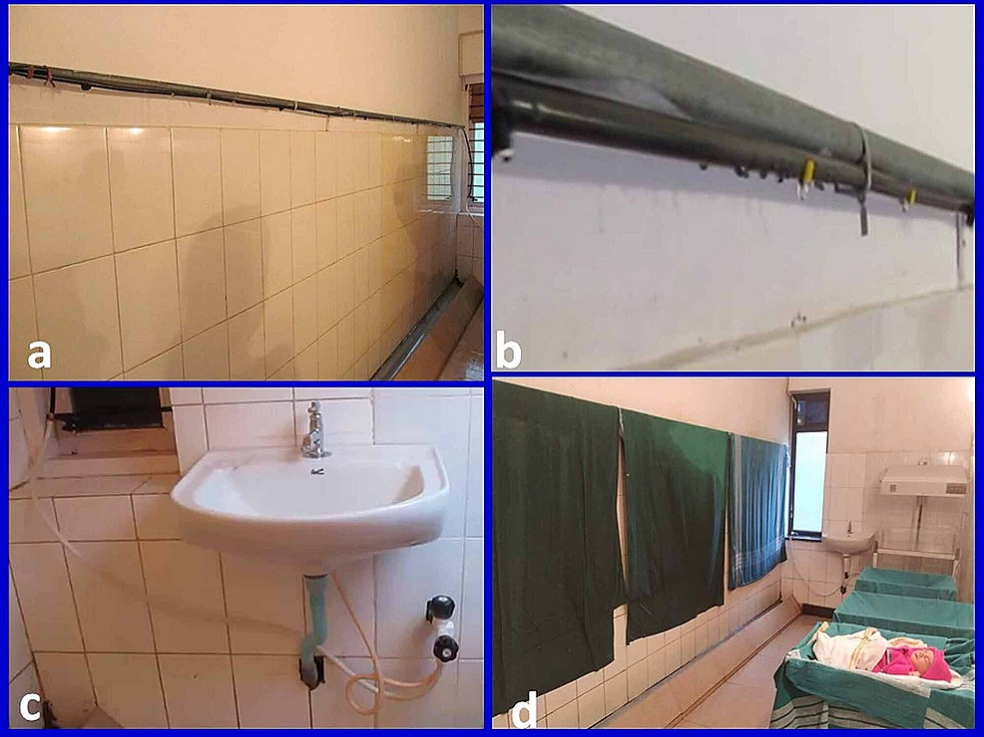
Humidifying system in the incubator room (a) drip system fixed on the wall; (b) dribbling water drops from drip pipe; (c) drip system connected to the tap; (d) wet cotton sheets hanging on drip pipe

In this descriptive interventional study, instead of an incubator box, we converted one entire room (14’/9’/10’) into an incubator (Video 1, Figure 1). Thirty preterm, LBW neonates delivered at different gestational ages were provided care in this incubator room. One or more babies could be managed simultaneously. We kept only one or two babies in the incubator room at a time and maintained a distance of 1-1.5 meters between babies to minimise chances of cross-infection. The room temperature was maintained at 32 °C, and room humidity was maintained at 70% (Figures 1, 2). All babies were made to wear woolen sweaters to prevent temperature loss. Babies’ temperature was recorded every three hours (Figure 1). A sponge bath with lukewarm water was given every morning. The hydration and nutrition needs of babies were met with IV fluids/nasogastric (NG) tube feeding with expressed breast milk (EBM) or formula feeds. Prophylactic antibiotics, ceftriaxone and amikacin injections, were given intravenously in adequate doses, twice daily. Oxygen saturation was monitored by pulse-oximetry whenever needed.

Maintenance of room temperature

**Figure 3:**
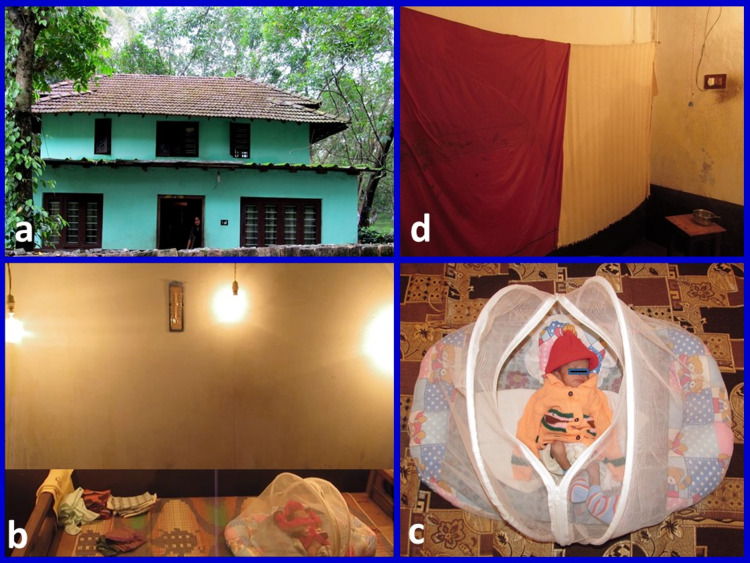
Original image of an incubator at home (a) the house of a below-poverty line mother; (b) three 200-watt bulbs can be seen hanging, and room thermometer is fixed on the wall; (c) the baby wearing a sweater on the cot; (d) wet cotton sheets can be seen hanging on the wall

**Figure 4:**
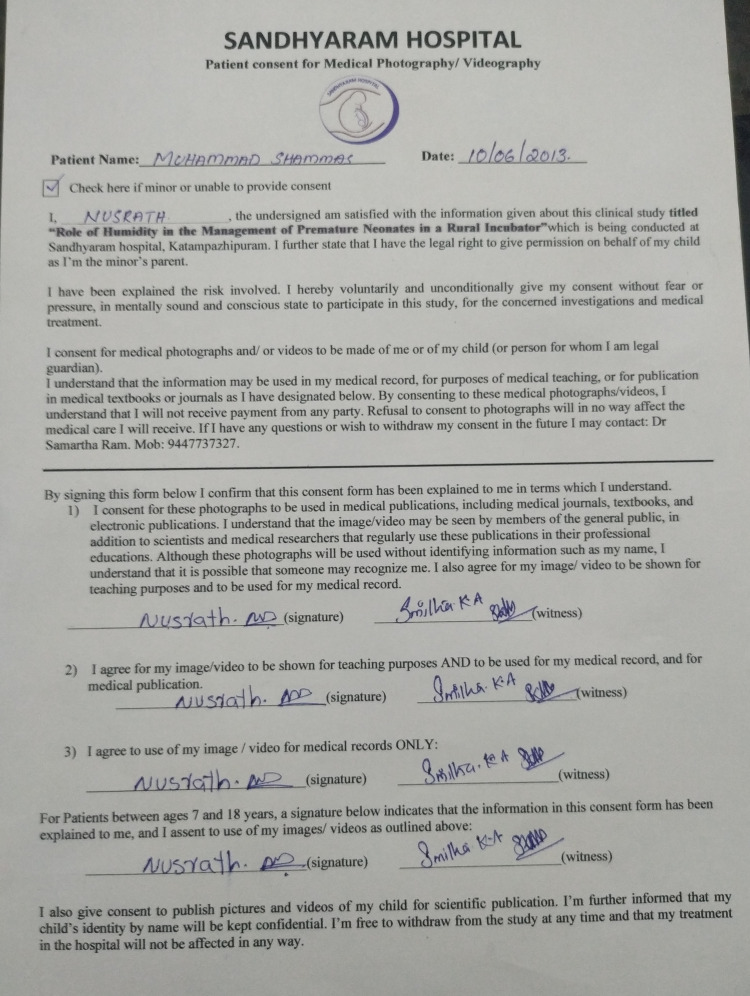
A sample of the consent form obtained from the participants in the study

Three 200-watt bulbs were fixed to the wall at a height of 1 meter from the babies. The room thermometer was fixed to the wall close to babies. When the temperature rose above 32 °C, one bulb was switched off. When the temperature fell below 32 °C, one bulb was switched on. In this way, the room temperature was maintained at around 32 °C consistently (Figures 1, 3).

**Video 1:** Incubator set-up

Maintenance of room humidity

Wet cotton sheets measuring 4’ × 6’ were spread on the opposite wall with the support of a stand (Figure 2). A drip system was connected to the running tap water to maintain wetness on the cotton sheets (Figure 2). A hygrometer was fixed to the wall near the babies to measure the humidity of the room (Figure 1). Cotton sheets were made wet by opening the tap when the humidity fell. The hygrometer reading would raise when the cotton sheets became wet, and the reading would fall when sheets became dry. The humidity of the room was maintained at 70-80% by turning the tap on or off as required (Video 2, Figure 2).

**Video 2:** Maintainance of room humidity

Meeting hydration and nutrition needs

Intravenous 10% dextrose with 1/5 normal saline was given through infusion pump in adequate doses to maintain hydration and nutrition. After stabilising the baby, NG tube feeding was added with EBM or infant formula feed.

We discharged the babies when they were stable and were able to be fed on breast/bottle. Before discharge, we made the parents create similar humidity and temperature settings at home so that the care could be continued by mothers (Video 3, Figure 3). 

**Video 3:** Incubator set-up at home

Method of feeding

After washing hands and taking aseptic precautions, an NG tube was introduced. Depending on the baby's birth weight, we gave a test feed of 3-5 cc of distilled water or saline. If there was no retching or vomiting, we continued feeding with EBM/infant formula feed. Every day, we would fix the volume of feed that had to be given for that particular day. Feeds were given at an interval of three hours. At the time of every feed, an NSG tube was introduced, the gastric contents were aspirated and measured. For example, if the fixed feed volume for that day was 10 ml, and if the aspirate volume was nil, a full 10-ml feed was given. On the other hand, If the aspirate volume was 3 ml, only the remaining 7-ml feed was given.

After every feed, the NSG tube was removed. Feed volume per feed for the next day was decided based on the aspirate volumes of the previous day. The feed volume was gradually increased provided the baby was digesting the feeds well. Feeding details were recorded in a chart (Figure 1C). Once the babies reached a stage of digesting 15-20 ml feeds, they were put on for breastfeeding. Some mothers did not secrete enough milk, and some LBW babies could not suck effectively at the breast. We gave bottle feeds (EBM/formula feeds) in such cases. These bottles were sterilised for every feed. At the time of every feed (three-hourly), the baby’s temperature, room temperature, room humidity, the volume of the stomach aspirate, and volume of the feed given were recorded and plotted on a chart (Figure 1C). Minimal necessary investigations were done based on clinical features.

The birth weight of each baby was recorded. For babies who developed neonatal respiratory distress syndrome (NRDS), the number of hours required for complete recovery and the number of hours that oxygen was given were recorded (Table 1). The incidences of babies developing septicaemia, necrotising enterocolitis (NEC), hypothermia, hypoglycemia, and hypocalcemia were recorded. The number of neonatal intensive care unit (NICU) admission days required and the number of home incubator days required with respect to each baby were documented (Table 1). Mode of delivery and reasons for LBW in each woman was also recorded. The number of neonatal deaths that occurred in the study period was noted (Table 1). Babies were followed up for two years to observe long-term morbidities like neurodevelopmental delay, impairment, cerebral palsy, and bronchopulmonary dysplasia. The results were compared with the findings in the literature.

Statistical analysis

Statistical analysis was performed using SPSS Statistics for Windows Version 20.0. (IBM, Armonk, NY). As the distribution was non-Gaussian, we presented the median with interquartile ranges for continuous variables.

## Results

Among the 30 neonates, the gestational weeks at birth ranged from 27 weeks + two days to 37 weeks, and the median value was 35 weeks + one day. Birth weights of babies ranged from 1 kg to 1.95 kg, and the median weight was 1.7 kg. Nine babies (30%) were very LBW (VLBW) babies with birth weights of less than 1,500 grams. Twenty-three babies (23/30, 74.1%) developed varying degrees of NRDS, and among them, 22 babies (95.6%) recovered with oxygen supplementation with a mask. Among these 22 babies, the duration of recovery ranged from four to 24 hours. Only one baby needed endotracheal tube intubation and ventilator support for four days. Four babies (4/30, 13.3%) developed septicaemia, and two of them developed NEC. One baby with NEC recovered, and the other baby died. None of the babies developed hypothermia, hypoglycemia, or hypocalcemia. Among the 29 survivors, NICU admission days ranged from five to 28 days.

After discharge, all the babies were cared for in in-home incubators, and the number of incubator days ranged from 15 to 60 days. The median value was 30 days. None of the babies faced any problem during home incubation. Among the 30 babies studied, 29 babies (96.6%) did well and survived as a result of this treatment (Table 1). On follow-up of the survivors for two years, we could not observe any neurodevelopmental impairment, cerebral palsy, or bronchopulmonary dysplasia.

Among the 30 women studied, 11 women (96.6%) had premature rupture of membranes (PROM), 19 women (96.6%) had scanty liquor, five women (96.6%) had pregnancy-induced hypertension (PIH), and eight babies (96.6%) had clinical features of intrauterine growth restriction (IUGR). Twenty-nine women (96.6%) underwent elective caesarean sections, and only one woman had a normal vaginal delivery.

**Table 1:** Clinical details and outcomes of neonates cared for in rural incubator CS: caesarean section; VD: vaginal delivery; GA: gestational age; NRDS: neonatal respiratory distress syndrome; NEC: necrotising enterocolitis; NICU: neonatal intensive care unit; NND: neonatal death; LBW: low birth weight; PROM: premature rupture of membranes; PIH: pregnancy-induced hypertension; IUGR: intrauterine growth restriction; FD: fetal distress

Serial no	Maternal age (years)	Mode of delivery	CRL GA [weeks + day(s)]	Birth weight (kg)	NRDS (yes/no: 1/0)	Duration of NRDS	No of hours of O_2_ given	Septicaemia (yes/no: 1/0)	NICU days	Days in home Incubator	NND (yes/no: 1/0)	Reason for LBW
1	22	CS	35 + 1	1.7	0	00	16	0	07	21	0	PROM
2	21	CS	32 + 0	1.5	1	06	30	0	12	36	0	PIH, IUGR
3	22	CS	37 + 0	1,9	0	00	10	NEC	04	00	1	Scanty liquor, IUGR
4	26	CS	32 + 2	1.2	1	06	36	NEC	21	60	0	Scanty liquor
5	28	CS	35 + 4	1.9	1	12	24	0	05	15	0	Scanty liquor
6	20	CS	31 + 2	1.1	1	16	24	0	28	58	0	Scanty liquor
7	23	CS	37 + 0	1.9	0	00	12	0	07	15	0	Scanty liquor, IUGR
8	27	CS	32 + 1	1.3	1	08	36	0	11	58	0	Scanty liquor, FD
9	32	CS	36 + 4	1.78	0	06	18	0	07	30	0	Scanty liquor, IUGR
10	27	CS	34 + 1	1.8	1	18	24	0	09	30	0	PROM
11	32	CS	34 + 4	1.9	1	08	12	0	06	28	0	PIH, scanty liquor
12	21	CS	35 + 1	1.7	1	10	24	1	06	28	0	Scanty liquor
13	18	CS	34 + 4	1.9	1	08	16	0	07	28	0	PROM
14	28	CS	33 + 1	1.3	1	20	36	1	17	70	0	Scanty liquor
15	27	CS	34 + 4	1.8	1	10	20	0	10	30	0	PROM
16	18	CS	34 + 3	1.9	1	04	12	0	09	28	0	PROM
17	38	CS	33 + 1	1.33	1	12	36	0	11	60	0	PIH, scanty liquor
18	32	CS	34 + 4	1.9	1	08	24	0	06	30	0	PIH, scanty liquor
19	28	CS	33 + 4	1’9	1	10	24	0	06	28	0	Scanty liquor, IUGR
20	26	CS	36 + 4	1.7	1	10	24	0	09	45	0	PIH, scanty liquor
21	22	CS	37 + 0	1.85	0	00	08	0	06	28	0	PROM
22	21	VD	36 + 6	1.95	1	06	12	0	10	30	0	Scanty liquor, IUGR
23	24	CS	36 + 0	1.75	1	12	36	0	08	30	0	PROM
24	19	CS	35 + 6	1.8	0	0	20	0	12	30	0	Scanty liquor, IUGR
25	23	CS	36 + 5	1.85	0	0	08	0	05	30	0	PROM, scanty liquor
26	23	CS	35 + 5	1.65	1	8	24	0	07	28	0	Twins
27	23	CS	35 + 2	1.5	1	10	24	0	07	28	0	Twins
28	30	CS	27 + 2	1.0	1	20	36	0	10	45	0	PROM
29	23	CS	36 + 5	1.65	1	05	24	0	10	28	0	PROM
30	22	CS	33 + 5	1.5	1	24	48	0	15	28	0	PROM, IUGR

## Discussion

PTB is one of the major causes of neonatal mortality and long-term morbidity [1]. A majority of preterm births occur in low-resource settings with suboptimal facilities [2]. As the costs and expertise involved in premature neonatal care are very high and scarce, many babies lose their lives in low-resource settings. Hence, there is an immediate need to devise and implement simpler and low-cost techniques to save these babies.

In the method we discussed in this study, we converted one entire room (14’/9’/10’) into an incubator. We used three 200-watt bulbs to maintain the room temperature at 32 °C. In case of power failure, the temperature of the room did not drop quickly as a large volume of air hat got warmed up. Also, we had given woolen sweaters to every baby to prevent heat loss. For these reasons, babies maintained adequate temperature very well without fluctuations. As the room was very spacious, we could manage more than one baby at a time.

We used wet cotton sheets to maintain the humidity of the room at around 70% [4]. This provided humidified air for breathing. The baby’s skin did not dry up and maintained good moisture and softness, which might have helped regulate the temperature better.

In our study, we had given NG tube feeds at an interval of three hours. At the time of every feed, an NG tube was introduced, and the feed was given. The tube was removed after the feed. We used thin and soft NG (F) tubes to avoid oesophageal/pharyngeal mucosal injuries [5]. This enabled us to avoid the use of a continuous indwelling catheter, which is often a source of infection and septicaemia. This also helped us avoid gastric bleeding, which can occur due to the constant irritation of gastric mucosa by the tip of the catheter.

Our ‘makeup volume feeds’ technique helped prevent abdominal overdistension, vomiting, and aspiration into the lungs. Bottle feeds were given with EBM/infant formula feeds only when babies did not have enough power to suck on the nipple or when mothers were not secreting enough milk. We used infusion pumps to give IV fluids, thereby preventing overhydration and pulmonary oedema.

In developing countries, the overall mortality rate due to NRDS is around 36.5% [7]. In babies requiring invasive ventilation, the mortality rate is around 62.7%. In contrast, it is just 2.2% with babies managed without invasive ventilation [5]. In our study, 23/30 babies (74.1%) developed NRDS. Only one baby needed invasive ventilation, and even that baby survived. The mortality was 0% in this NRDS subgroup. The effect of humid, warm breathing air on lung alveoli could be the reason for these excellent results. 

Neonatal sepsis is the third leading cause of neonatal mortality in developing countries. Infants with sepsis are nearly three times more vulnerable to death when compared to infants without sepsis [8,9]. The condition is responsible for 13% of all neonatal deaths, and 42% of these deaths occur in the first week after birth. Up to 20% of all VLBW infants die because of sepsis [10-12]. In our study, we used an open caring system, and we had neonatal sepsis in four out of 30 (12.9%) babies. We had only one (2.3%) neonatal death. The open caring system, avoidance of continuous indwelling NG tubes, strict hand-washing, minimum handling, and minimal investigations by avoiding multiple needle pricks could be the reasons for fewer sepsis cases in our study. We had kept only one or two babies (with a minimum distance of 1.5 meters between them) in the room at any given point in time. This prevented cross-infection. The only baby we lost died due to septicaemia with NEC. The mother of this baby had antenatal chickenpox, and the baby had chickenpox skin lesions at birth. 

Birth weights of babies in our study ranged from 1 kg to 1.95 kg, with a median weight of 1.7 kg. The majority of babies had growth restrictions with scanty liquor (Table 1). This could be the reason for the values being far below the expected weight. In spite of this, our humidity intervention had very good outcomes.

There were nine (30%) VLBW babies in our study population. Survived VLBW infants are at an increased risk of developing morbidities like neurodevelopmental impairment, cerebral palsy, and bronchopulmonary dysplasia [13-15]. On following up with these babies for two years, we could not observe any of these morbidities.

In our study, 29/30 women (96.6%) underwent caesarean deliveries. We preferred elective caesarean sections to avoid the stress of labour on already sick and LBW babies.

Hospital stay

Prolonged hospital stay is very common with VLBW babies. We discharged these babies when they were stable and were able to feed on breast milk or formula feeds. We made the parents create a similar set-up of humidity and temperature (with wet cotton sheets and bulbs) at home before discharge. We provided basic training to mothers to carry out further care of their babies at home. None of the babies died, and we managed to avoid extended hospital stays and high costs.

Limitations and future trends

The study was conducted at a small hospital with a small sample size. There was no comparative group to prove the method's efficacy. Hence, further randomised controlled studies with larger sample sizes, which would compare our results with those of babies cared for in conventional incubators, are needed to validate our method and prove its efficacy.

## Conclusions

Open nursing of functionally premature, LBW neonates at room temperature of 32 °C and humidity of 70% is highly cost-effective and can result in excellent outcomes. This method is easily practicable in low-resource settings and even at home. It can significantly bring down NICU admission days, hospital costs, and is ideal for caring for neonates in developing countries.
